# Supplementary material for: Simple anthropometric measures to predict visceral adipose tissue area in middle-aged Indonesian men
Source: PLoS One. 2023 Jan 6;18(1):e0280033. doi: 10.1371/journal.pone.0280033 (PMC9821461; doi:10.1371/journal.pone.0280033)
Supplement: Attachment — Submitted filename: Response to Reviewers.docx [file pone.0280033.s002.docx]

1. Please use updated reference in line 75. This data is from 2016.

Thank you for the suggestion. We have added new information from the newest reference regarding the prevalence of obesity in line 52-55.

2. Please check if the reference is correct in line 98.

Thank you for the correction. We have re-checked the reference and made a correction to the reference that we cited. (Reference no. 13)

3. In line 98, the author said Indonesia is a country with more than 600 ethnicities, and then in line 117, all the volunteers were recruited from one university. What’s the ethnic diversity of this group? Will it be a good representative group for all Indonesian population?

Thank you for the comment. The Cipto Mangunkusumo National General Hospital is the central referral hospital in Indonesia, with patients coming from all over the country. We have added this information to line 95.

4. Please insert the sample size in line 116 and what analysis method did you use for this sample size, Power analysis?

Thank you for the reminder. For the sample size, we used the general formula for calculating total sample size when using the correlation coefficient (Hulley et al, 2013)^1^ as follows:

$$N=\left[ \frac{\left( Z_{\alpha}+Z_{\beta} \right)}{0.5\ln\left( \frac{1+r}{1-r} \right)} \right]^{2}+3$$

We used 95% Confidence Interval for type I error ($Z_{\alpha}$). For the type II error ($Z_{\beta})$, we used 10%. According to a previous study by Camhi et al (2011)^2^, the correlation coefficient (*r*) between VAT and waist circumference was 0.73 – 0.77, whereas the r between VAT and BMI was 0.61 – 0.69. We used the table from Hulley et al (2013) and obtained the minimum sample size ($N$) of 25.^1^ In this study, our sample size was 32 which is larger than the minimum sample size required.

We have added information on our sample size calculation in line 99-105.

References:

1. Hulley SB. Designing Clinical Research. 5^th^ edition. Philadelphia: Lippincott Williams & Wilkins. 367 p.

2. Camhi SM, Bray GA, Bouchard C, Greenway FL, Johnson WD, Newton RL, et al. The Relationship of Waist Circumference and BMI to Visceral, Subcutaneous, and Total Body Fat: Sex and Race Differences. Obesity (Silver Spring). 2011 Feb;19: 402-8.

5. In line 137 normoweight? or normal weight?

Thank you for the comment. We have corrected normoweight to healthy weight in line 154 and 178, as this is the correct terminology for what we meant by normoweight (normal weight). (line 131 and 153)

6. Please insert the reference paper in line 151.

Thank you for the comment. We have added the reference to the cut-off value of Visceral Adipose Tissue (VAT) area for determining central obesity, which is Reference No. 21.

7. Why did you choose 40-60 as the age limit of the volunteers? Why not age 20 plus? line 157 nornoweight?

Thank you for the question. Our decision to not include participants under the age of 40 is because, according to a meta-analysis by Wong et al (2020)^1^, the prevalence of central obesity for men is higher in subjects aged > 40 years. This research did not include men aged >60 years because in Indonesia they are categorized as geriatric patients^2^. Moreover, according to research by Schousboe et al (2018)^3^, central obesity and VAT are not associated with atherosclerotic cardiovascular disease events in men aged more than >65 years.

Source:

1. Wong MCS, Huang J, Wang J, Chan PSF, Lok V, Chen X, et al. Global, regional and time‑trend prevalence of central obesity: a systematic review and meta‑analysis of 13.2 million subjects. Eur J Epidemiol. 2020; 35:673-83.
2. Republic of Indonesia. Law of the Republic of Indonesia number 13 in 1998 concerning elderly welfare. Jakarta (ID): Republic of Indonesia; 1998. 24 p.
3. Schousboe JT, Kats AM, Langsetmo L, Vo TN, Taylor BC, Schwartz AV. Central Obesity and Visceral Adipose Tissue Are Not Associated With Incident Atherosclerotic Cardiovascular Disease Events in Older Men. J Am Heart Assoc. 2018;7:e009172.

We have corrected normoweight to healthy weight. (line 131 and 153)

8. It would be good to put in height and weight in table 1. So we know the body weight range for the people. The reason is because one limitation of conducting CT scan is its inapplicability for extremely obese people. 68.8% of the subjects in this study were overweight or obese.

Thank you for the suggestion. We have added height and weight in Table 1.

9, In figure 1, please note what is the significant cut point of P value in legend.

Thank you for the suggestion. We have added the cut-off point for p value in legend.

10. Figure 2 didn’t mark A,B in the plot.

Thank you for the reminder. We have marked (A) and (B) in the plots.

11. In line 183-193, this paper suggested using WC or BMI for central obesity, how about fat free mass? Have you considered adding FFM as an factor that influences WC or BMI? I suggest that you should adjust your WC or BMI by FFM and then do the regression with VAT again.

We have considered that fat-free mass might affect WC and BMI in predicting obesity. However, according to research by Górnicka et al (2022), there were relatively strong correlations between waist circumference and fat-mass (r=0.847) and fat-free mass (r=0.655). BMI also had a strong correlation with fat mass (r=0.939) and fat-free mass (r=0.529). Therefore, we decided to use BMI and waist circumference as a predictor for central obesity, as they can also be conducted with simpler measurement instruments.

Reference:

1. Górnicka M, Szewczyk K, Białkowska A, Jancichova K, Habanova M, Górnicki K, et al. Anthropometric Indices as Predictive Screening Tools for Obesity in Adults; The Need to Deﬁne Sex-Speciﬁc Cut-Off Points for Anthropometric Indices. Appl. Sci. 2022;12:6165.

12. In line 196, same as comment 3, is this population sample a good representative for all of Indonesia?

Thank you for the question. The subjects of this study were recruited using time-location sampling as a form of multi-step sampling procedure (Leon et al, 2015). The time of sampling was December 2018 to February 2019, whereas the location was the Cipto Mangunkusumo National General Hospital. The location is representative of Indonesian population because it is a national referral hospital. The method of time-location sampling can be non-biased if we considered frequency of venue attendance (Leon et al, 2015). In this study, we considered FVA by including all respondents who were willing to participate during the time of sampling at the Cipto Mangunkusumo National General Hospital.

We have added information on the sampling method in line 107-108.

Reference:

1. Leon L, Jauffret-Rouside M, Strat YL. Design Design-based inference in time-location sampling. Biostatistics. 2015;16:565-79.

Reviewer #2: The purpose of this study is to develop a formula to predict visceral adipose tissue (VAT) area from waist circumference (WC) and BMI and to determine its cutoff value. There have been very few papers on the evaluation of visceral fatty obesity using CT or MRI in Indonesia, and the method of estimating it from abdominal circumference and BMI is a simple and very interesting study. On the other hand, the very small number of subjects (small sample size and male only) is clearly a weak point of this study. In addition, there are many papers examining the relationship between visceral fat and body composition using CT or MRI, but I could not understand why a simple method that does not correct for age, weight, etc. needs to be developed. In addition, considering the large number of previous international studies, this study should have a much larger number of cited papers.

We thank you for the comments and interest on this study.

1. As for the sample size, we used the general formula for calculating total sample size when using the correlation coefficient^1^ as follows:

$$N=\left[ \frac{\left( Z_{\alpha}+Z_{\beta} \right)}{0.5\ln\left( \frac{1+r}{1-r} \right)} \right]^{2}+3$$

We used 95% Confidence Interval for type I error ($Z_{\alpha}$). For the type II error ($Z_{\beta})$, we used 10%. According to a previous study by Camhi et al (2011)^2^, the correlation coefficient (*r*) between VAT and waist circumference was 0.73 – 0.77, whereas the r between VAT and BMI was 0.61 – 0.69. We used the table from Hulley et al (2013) and obtained the minimum sample size ($N$) of 25.^1^ In this study, our sample size was 32 which is larger than the minimum sample size required. We have added information on our sample size calculation in line 99-106.

2. We conducted this research only on males because the risk of fatality caused by obesity is more significant in males compared to females. This can be observed by the life expectancy of obese males which is significantly less than that of obese females (Nagai et al, 2015).^3^ We have added this information in line 64-65.
3. Regarding the age of our samples, we decided to include only males aged 40-60. According to a meta-analysis by Wong et al (2020)^4^, the prevalence of central obesity for men is higher in subjects aged > 40 years. This research did not include men aged >60 years because in Indonesia they are categorized as geriatric patients.^5^ Moreover, according to research by Schousboe et al (2018)^6^, central obesity and VAT are not associated with atherosclerotic cardiovascular disease events in men aged more than >65 years. We have added our reason in line 65-67.
4. As for the sampling technique, the subjects of this study were recruited using time-location sampling as a form of multi-step sampling procedure (Leon et al, 2015)^7^. The time of sampling was December 2018 to February 2019, whereas the location was the Cipto Mangunkusumo National General Hospital. The location is representative of Indonesian population because it is a national referral hospital. The method of time-location sampling can be non-biased if we considered frequency of venue attendance (Leon et al, 2015)^4^. In this study, we considered FVA by including all respondents who were willing to participate during the time of sampling at the Cipto Mangunkusumo National General Hospital. We have added information on the sampling method in line 107-108.
5. As for why this study was conducted in a limited sample size on a specific sex, age, and recruited samples using the TLS method, it is because the cost of CT scan is expensive, which is $200-600 per subject. We decided to use the aforementioned study design because we had a relatively limited research budget.
6. Below are the additional references:
   1. Hulley SB. Designing Clinical Research. 5^th^ edition. Philadelphia: Lippincott Williams & Wilkins. 367 p.
   2. Camhi SM, Bray GA, Bouchard C, Greenway FL, Johnson WD, Newton RL, et al. The Relationship of Waist Circumference and BMI to Visceral, Subcutaneous, and Total Body Fat: Sex and Race Differences. Obesity (Silver Spring). 2011 Feb;19: 402-8.
   3. Nagai M, Kuriyama S, Kakizaki M, Ohmori-Matsuda K, Sone T, Hozawa A, et al. Impact of obesity, overweight and underweight on life expectancy and lifetime medical expenditures: the Ohsaki Cohort Study. BMJ Open. 2012;2:e000940.
   4. Wong MCS, Huang J, Wang J, Chan PSF, Lok V, Chen X, et al. Global, regional and time‑trend prevalence of central obesity: a systematic review and meta‑analysis of 13.2 million subjects. Eur J Epidemiol. 2020; 35:673-83.
   5. Republic of Indonesia. Law of the Republic of Indonesia number 13 in 1998 concerning elderly welfare. Jakarta (ID): Republic of Indonesia; 1998. 24 p.
   6. Schousboe JT, Kats AM, Langsetmo L, Vo TN, Taylor BC, Schwartz AV. Central Obesity and Visceral Adipose Tissue Are Not Associated With Incident Atherosclerotic Cardiovascular Disease Events in Older Men. J Am Heart Assoc. 2018;7:e009172.
   7. Leon L, Jauffret-Rouside M, Strat YL. Design Design-based inference in time-location sampling. Biostatistics. 2015;16:565-79.

Major comments
The importance of studies participated in men should be added to Line73-86. Similarly, the importance of assessing visceral fat should be added to Lines 91-94.

Thank you for the suggestion. We have added the importance of assessing obesity in men in line 64-67, and the importance of assessing visceral fat to line 71-77.

It should be emphasized that most of the previous studies in Indonesia used simple methods such as the impedance method, and few studies have used in CT or MRI of high-precision method. The following paper assessing visceral fat in Indonesia may be helpful to cite.
doi: 10.1016/j.orcp.2020.11.003.
doi: 10.3390/jcm7050096
doi: 10.1016/j.diabres.2007.01.062.
doi: 10.2337/dc18-1074.

Thank you for the suggestion. We have added the information and cited the papers above in line 217-223.

Line 116 Were the subjects recruited from patients attending the hospital? Please add the method of recruiting subjects.

Thank you for the question. The subjects of this study were recruited using time-location sampling as a form of multi-step sampling procedure (Leon et al, 2015). The time of sampling was December 2018 to February 2019, whereas the location was the Cipto Mangunkusumo National General Hospital. The location is representative of Indonesian population because it is a national referral hospital. The method of time-location sampling can be non-biased if we considered frequency of venue attendance (Leon et al, 2015). In this study, we considered FVA by including all respondents who were willing to participate during the time of sampling at the Cipto Mangunkusumo National General Hospital.

We have added this information in line 107-108.

Reference:

1. Leon L, Jauffret-Rouside M, Strat YL. Design Design-based inference in time-location sampling. Biostatistics. 2015;16:565-79.

Line 125 and Line 134. What conditions were body composition and CT assessments measured? Please describe the measurement conditions, such as clothing, fasting time, etc.

Thank you for the question. During the measurement, the samples used standard robes for CT scan and had fasted for 4 hours prior to the measurement. We now have included this information in line 116-118.

Line 131. Please add a citation to show the rationale for setting the HU values for fat in the range of -190 to -30 HU.

Thank you for the comment. We have added the references which are References No. 17-20.
