# Supplementary material for: Simple anthropometric measures to predict visceral adipose tissue area in middle-aged Indonesian men
Source: PLoS One. 2023 Jan 6;18(1):e0280033. doi: 10.1371/journal.pone.0280033 (PMC9821461; doi:10.1371/journal.pone.0280033)
Supplement: Attachment — Submitted filename: Response to Reviewers.docx [file pone.0280033.s003.docx]

1. Line 29 It should be changed to "The diagnosing of central obesity...".

Thank you for the correction. We have revised Line 29 to “The diagnosing of central obesity...”

2. Line 131 It should be changed from “healthy weight” to “normal weight”

Thank you for the correction. We have changed the term “healthy weight” to “normal weight” in Line 132 and 154.

3. The percentage of overweight or obese persons in this study (68.8%) appears to be higher than the actual situation in Indonesia. Therefore, the results obtained in this study may not be generalizable. This point should be added to the limitations.

Thank you for the suggestion. We have added this limitation in Line 236-239:

*“It is also important to note that the percentage of people with obese or overweight in this study is higher than the actual situation in Indonesia. Therefore, this study’s results might not be generalizable.”*
